# A Rare Case of Therapy-Related B-cell Acute Lymphoblastic Leukemia Arising From Acute Myeloid Leukemia

**DOI:** 10.7759/cureus.45745

**Published:** 2023-09-21

**Authors:** Vishvaas Ravikumar, Jacob Berkowitz, Omar Khan, Diana P Garcia, Ramalingam Ratnasabapathy

**Affiliations:** 1 Internal Medicine, Oregon Health & Science University (OHSU), Portland, USA; 2 Internal Medicine, Kirk Kerkorian School of Medicine at University of Nevada, Las Vegas (UNLV), Las Vegas, USA; 3 Pathology, Aurora Diagnostics LMC Pathology Services, Las Vegas, USA; 4 Pathology, Quest Diagnostics, Las Vegas, USA; 5 Hematology and Oncology, Comprehensive Cancer Centers of Nevada, Las Vegas, USA

**Keywords:** neutropenic sepsis, next generation sequencing (ngs), therapy-related leukemia, acute myeloid leukemia (aml), acute lymphoblastic leukemia (all)

## Abstract

Therapy-related acute lymphoblastic leukemia (t-ALL) is a rare potential complication of chemotherapy. We describe the case of a 47-year-old male patient who was originally diagnosed with t(8;21) positive acute myeloid leukemia (AML) in 2019, received chemotherapy, achieved remission, and was disease-free for the next two years. During a routine follow-up in 2022, he was found to have developed subclinical pancytopenia, and further studies indicated a diagnosis of pH-negative, near-tetraploid B-cell acute lymphoblastic leukemia (B-ALL) that was positive for a Tier 1 TP53 mutation, consistent with t-ALL. The patient had a prolonged treatment course complicated by social factors, such as the impact of both disease and treatment on his ability to work enough to make a living and live life with the quality he desired. The patient elected to pause treatment and resume it at a later date, after which, unfortunately, significant disease progression occurred and the patient died from complicating neutropenic sepsis and variceal bleeding. This case illustrates the challenges of managing social circumstances and patient goals in the setting of medically necessary but potentially harsh treatment courses. Given the aggressive nature of t-ALL and its overall poor prognosis, goals of care must be re-evaluated and discussed often to ensure alignment of therapy with a patient’s wishes.

## Introduction

Acute leukemia is a cancer of the blood and bone marrow defined as an uncontrolled proliferation of immature white blood cells. The various types of leukemia are classified based on the lineage of the malignant precursor cells, myeloid or lymphoid. Acute myeloid leukemia (AML) and acute lymphoblastic leukemia (ALL) are commonly seen pathologies in the literature. However, instances of single patients acquiring both leukemia subtypes are quite rare. Exceedingly rare is the development of ALL secondary to chemotherapy, or therapy-related ALL (t-ALL) [1,2], in contrast to therapy-related AML (t-AML) or myeloproliferative disorders, which have been well-characterized and represented in the literature to date [3]. In this case report, we will describe the case of a patient who presented with AML, completed treatment, achieved remission, and was then diagnosed two years later with novel B-cell acute lymphoblastic leukemia (B-ALL), with additional workup such as next-generation sequencing (NGS) pointing towards t-ALL specifically.

This article was previously presented as a meeting abstract at the 2023 American College of Physicians (ACP) Internal Medicine Meeting on April 29, 2023.

## Case presentation

**Figure 1:**
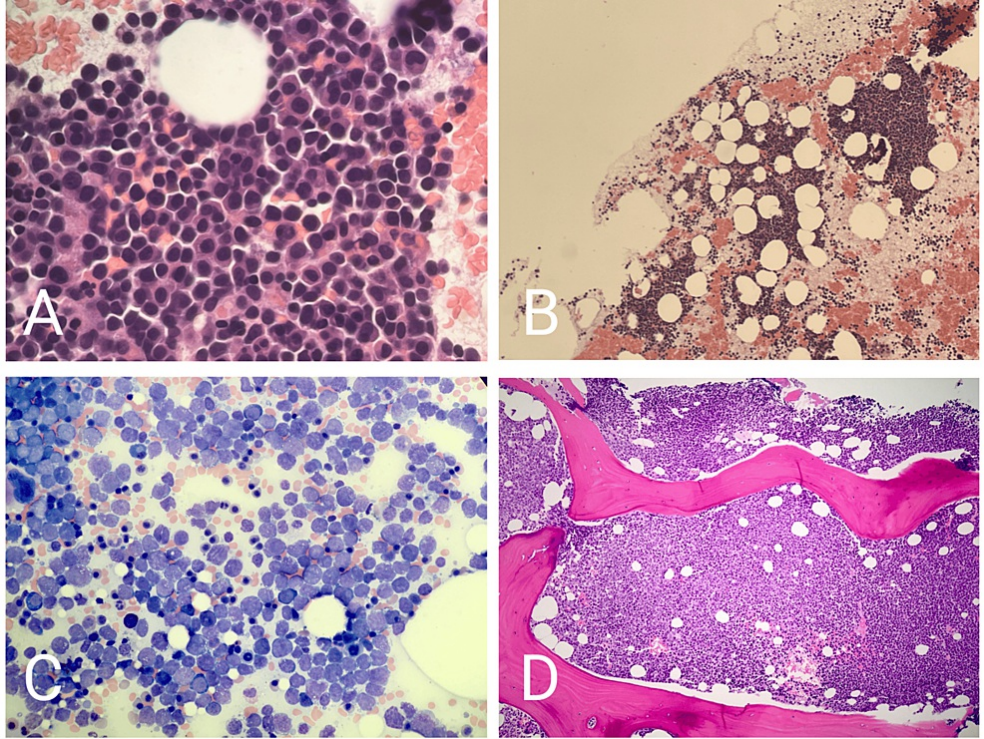
Comparison of the initial bone marrow core biopsy in 2019 (A, B) to the aspirate smear (C) and core biopsy (D) in 2022. A and B are hypercellular (70%) and comprised predominantly of leukemic blasts (80%). C and D are relatively more hypercellular (95%), with predominantly leukemic blasts (68%). Flow cytometry findings associated with each bone marrow sample suggested that the blasts depicted in A and B were of myeloid origin and those in C and D were of lymphoid origin.

Our patient was a 47-year-old male with a medical history of type II diabetes mellitus and gout, both well-controlled, as well as alcohol use disorder, who initially presented in 2019 to a community hospital emergency department with a fever, headache, and petechial rash. He was found to be severely pancytopenic, and a prompt bone marrow biopsy was performed. The findings were concerning for AML, showing 80% blasts in 70% hypercellular marrow (Figures 1A, 1B).

Flow cytometry indicated positivity for CD45 (dim), CD34 (99%), CD117 (94%), human leukocyte antigen-DR isotype (HLA-DR) (100%), CD13 (98%), CD33 (39%), CD56 (99%), and cMPO (81%). The karyotype of the patient showed 45X, -7, del(5), t(8;21)(q22;q22.1), del(9), and the fluorescence in situ hybridization (FISH) analysis was found to be positive for t(8;21), RUNX1-RUNX1T1 mutations. The patient’s next-generation sequencing (NGS) panel was negative. With a confirmed diagnosis of AML, the patient was started on 7+3 induction therapy with daunorubicin and cytarabine. This initial round of therapy was complicated by severe hyperbilirubinemia; additionally, the patient’s bone marrow continued to show residual blasts post the initial induction. As a result, he required re-induction with 5+7 therapy, after which a follow-up bone marrow biopsy showed no immunophenotypic evidence of residual leukemic blasts, and the patient achieved remission. 

Over the next several months, the patient underwent three cycles of high-dose cytarabine (Ara-C) consolidation therapy, complicated by several prolonged hospital admissions secondary to peripherally inserted central catheter (PICC)-line-induced sepsis, neutropenic fevers, and continued hyperbilirubinemia. Despite these setbacks, the patient completed consolidation successfully by the end of 2019. Given the favorable risk karyotype of the patient’s AML, he was not offered stem cell transplantation at this time and was instead periodically monitored for the next two years, with unremarkable labs and a bone marrow biopsy during that period.

At a routine clinic visit in 2022, the patient was once again found to be progressively pancytopenic in the absence of identifiable clinical symptoms. This initially prompted concerns over the recurrence of the patient’s AML. However, a bone marrow biopsy instead suggested acute leukemia consistent with a B-lymphoblastic lineage, showing 68% marrow blasts in 95% hypercellular marrow (Figures 1C, 1D). Flow cytometry showed 64% B-lineage lymphoblasts, positive for CD19, HLA-DR, CD10, CD34, CD38, intracellular terminal deoxynucleotidyl transferase (TdT), and cytoplasmic CD22. Significantly, no co-expression of any myeloid-associated antigens or myeloperoxidase was noted. His real-time reverse transcription-polymerase chain reaction (RT-PCR) was negative for the AML1/ETO t(8;21) chromosomal translocation product, and FISH was now negative for the RUNX1-RUNX1T1 fusion, showing instead near-tetraploidy. Of note, the patient denied any significant family history of malignancies or environmental hazards at home, and having worked as a bartender for many years, he denied any occupational exposures that may have contributed to the rapid development of a second leukemia.

Following this new diagnosis, the patient was referred to a tertiary cancer center for additional workup and treatment. The presence of a Philadelphia-negative near-tetraploid B-cell ALL was confirmed, and furthermore, NGS indicated the presence of a Tier 1 TP53 mutation associated with t-ALL. The patient was started on hyper-CVAD (cyclophosphamide, vincristine, doxorubicin, and dexamethasone) A/B therapy for induction, with plans for future intrathecal chemotherapy for central nervous system (CNS) prophylaxis. A bone marrow biopsy performed on admission showed 34% B-lymphoblasts, immunophenotypically positive for CD19, CD22, CD24, CD34, CD38, Tdt, and HLA-DR. A low level of myeloperoxidase (MPO) was also seen, thought likely to be a reactive myeloid population versus a mixed blast phenotype. The patient’s clinical course was complicated by an acute-on-chronic subdural hematoma, which was simultaneously evacuated while the neurosurgery team placed an Ommaya reservoir for intrathecal access. Following the completion of one cycle of hyper-CVAD therapy, the patient achieved clinical remission of his B-ALL. However, while several more cycles of hyper-CVAD were planned, the patient declined further treatment at the time due to side effects and interference with his ability to work and make a living.

The patient presented to a local community hospital once again in late 2022 with a fever and malaise. He had not received any treatments since achieving remission of his B-ALL and had had no follow-up since discontinuing treatment. He was found to be once again severely pancytopenic. Due to the insufficient leukocyte count in his bone marrow, flow cytometry was performed on peripheral blood instead and showed 36% blasts with matching flow cytometry findings from his earlier B-ALL. Interestingly, however, neogenomics performed on the bone marrow indicated the cancer was positive for CD10, CD19, CD22, Pax5, CD34, Tdt, and a new finding of MPO, while being negative for CD13, CD33, and CD117.

**Figure 2:**
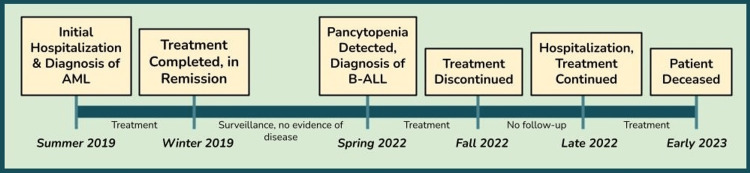
A timeline summary of the case from initial diagnosis of AML in 2019 to patient death during treatment of t-ALL in 2023 AML: acute myeloid leukemia; t-ALL: therapy-related acute lymphoblastic leukemia

The patient initially left against medical advice for personal reasons but returned within a month for re-admission and treatment. He was started on mini-CVD (cyclophosphamide, vincristine, and dexamethasone) and inotuzumab therapy. While his cancer was responsive to treatment and arrangements had been made for bone marrow transplantation in the near future, the patient acutely began to develop a neutropenic fever that evolved into sepsis, as well as significant upper gastrointestinal variceal bleeding. While being managed for these complications, the patient developed sudden cardiac arrest and passed away despite attempts at resuscitation. The patient’s disease and treatment course are summarized in Figure 2.

## Discussion

When a patient is diagnosed with leukemia, they are categorized as having either myeloid-lineage-derived disease or lymphoid-lineage-derived disease, based on which immunophenotypic markers are present on or within the cells. Mixed-phenotype acute leukemias (MPAL) display cytochemical or immunophenotypic features distinctive of both lineages, are characterized as biphenotypic in nature, and have a reported incidence of 1%-5% of acute leukemias [4]. Rare are bilineal leukemias, where two distinct clonal populations are present in one patient at the same time [5], and sequential presentations of different leukemias, whether occurring via one leukemia lineage switching into another or clinically distinct secondary leukemia developing sometime after the first is diagnosed and treated [1,2]. 

Neither the patient’s initial AML nor the B-ALL diagnosed later had features suggestive of biphenotypic leukemia, and no bilineal population was detected during his first disease course. It is well reported in the literature that prior chemotherapy can lead to myelodysplastic syndromes, or AML [3]. As reported in a study by Rosenberg et al. (2017), evidence suggests that among all patients who undergo medical treatment for cancer, those who have received prior genotoxic treatment modalities have an increased incidence of leukemia after treatment compared to the general population, with a standardized incidence ratio (SIR) of 1.62 CI (1.45-1.79) [1]. In patients with prior hematologic malignancies, the SIR associated with the development of ALL is reported as high as 5.57 CI (4.38-6.78) [1]. In a multicenter study by Saygin et al. (2019), it is reported that the latency period between genotoxic therapy exposure and diagnosis of malignancy is a median of five years for both t-ALL and t-AML [2], a longer period than what was observed with this patient's approximately two-year latency period. Exposure to topoisomerase II agents is associated with a shorter latency period to the diagnosis of t-ALL (median, 4.7 years; range, 0.5-16) when compared to all t-ALL patients who did not receive this therapy (median, 6.3 years; range, 1-35) [2]. However, this would not explain the shorter latency period observed in this patient's case given the lack of topoisomerase II agent use. Of the t-ALL cases reviewed in Saygin et al. (2019), 63% received alkylating agents, 33% received topoisomerase II inhibitors, 30% received both of these therapies, and only 11% had received other forms of chemotherapy [2]; our patient received the anthracycline daunorubicin and the anti-metabolite cytarabine for therapy of his AML, which would place him in the 11% of cases that developed t-ALL from agents such as these.

Reports of t-ALL are much rarer than t-AML, but based on the limited genetic evidence available at present, certain features associated with t-ALL were seen in this patient's malignancy. Specifically, the presence of a single Tier 1 TP53 mutation, as well as the detection of myeloid features in the finding of MPO on the patient’s most recent neogenomics test, were both observed in this patient’s second cancer and also seen in other cases of t-ALL [2]. Other specific gene mutations, such as those in MLL, DNMT3A, RUNX1, ASXL1, CDKN2A, IKZF1, FANCL, and FANCD2, have been seen in other cases of t-ALL [1,2], but were not detected in this patient's suspected t-ALL. These data are limited to a small cohort of patients at this time, and further studies are needed in the future to further define the genetic features of t-ALL. At present, there are no prognosis-related outcomes tied to these mutations in the context of t-ALL.

When findings of lymphoid and myeloid disease are both present, a diagnosis of MPAL may be suspected. In this case, while MPO was detected on the final neogenomics test, this finding without other accompanying myeloid features is insufficient for a diagnosis of MPAL when using the European Group of Immunological Characterization of Leukemia (EGIL) classification [6]. The most recent 2016 revision of the World Health Organization (WHO) guidelines also recommends caution in this scenario given that otherwise typical B-ALL can express low-level myeloperoxidase without other features of myeloid differentiation, and there is uncertain clinical significance to this finding [7]. In this case, management would not change whether this patient's diagnosis was B-ALL or MPAL B/myeloid, involving ALL-like induction therapy followed by an allogeneic stem cell transplant [6], as was in progress for this patient.

Neutropenic fever and ensuing sepsis are major risks in the setting of significant immunocompromise caused by both leukemia and the chemotherapies used to manage it [8]. Bleeding is also a reported complication of acute leukemias through associated thrombocytopenia, with potential contribution from intensive induction therapy [9] and further exacerbation by prior extensive alcohol use in this patient. Developing methods to identify patients at high risk for bleeding prior to treatment may improve overall survival outcomes, as described in a recent study by Versluis et al. (2022) in the setting of intensive induction therapy for AML [9]. While discontinuing therapy following the patient’s initial B-ALL diagnosis was due to the patient’s expressed wishes, the gap in treatment led to significant clinical deterioration and worsening of the patient’s prognosis by the time treatment resumed.

## Conclusions

The development of t-ALL is a rare potential complication of prior chemotherapy with poorer outcomes than non-therapy-related ALL. Management involves induction therapy followed by allogeneic stem cell transplantation for consolidation. Treatment proved challenging in this case due to the continued evolution of the disease, complications that arose during therapy, and social factors that interrupted the indicated treatment course. Consideration of all these factors is important when approaching rare and aggressive cancers. 
